# A Qualitative Vignette Study of Perceived Barriers and Facilitators of Bystanders in Racism in the Higher Education Context in Finland

**DOI:** 10.1177/08862605251315776

**Published:** 2025-01-28

**Authors:** Minna Lyons, Viivi Mäkinen, Raheemah Arogundade, Tuomas Zacheus

**Affiliations:** 1 Liverpool John Moores University, UK; 2 University of Helsinki, Finland; 3 University of Wolverhampton, Birmingham, UK

**Keywords:** bystander, barriers and facilitators, racism, Finland, university, qualitative

## Abstract

Bystanders play a potentially important role in intervening in incidents of racism, but they often fail to act. Much research has focused on investigating facilitators and barriers to bystander behavior, but mainly in the context of sexual violence. There is a dearth of research in the context of racism, especially outside the English-speaking world. In this pilot study, we employed a qualitative approach to explore bystander facilitators and barriers in higher education students in Finland. Online participants (*N* = 649) read two vignettes depicting a racist incident and wrote open-ended answers on factors that would facilitate and prevent intervention. We constructed six main themes using an inductive thematic analysis: (i) Perceived self-efficacy to intervene; (ii) Justification and moral reasoning; (iii) Clarity of the situation; (iv) Responsibility and permission to act; (v) Social relationships, support, and presence of others; and (vi) Consequences and impacts of action. We discuss the results with a reference to theories and literature, as well as the unique context of Finland.

## Introduction

Bias-based bullying and discrimination based on ethnicity/racialized identities have been well-recorded in higher education institutions, both at institutional and individual levels (e.g., Morrison et al., 2023; Osbourne et al., 2021, 2023; Wong et al., 2021; Young et al., 2015). Although racism in higher education settings can be visible, and easily recognizable (Moore & Bell, 2017), this type of bullying often occurs in a covert form (e.g., Ogunyemi et al., 2020), for example, as subtle slurs or jokes that are more difficult to tackle than overt, aggressive actions. Being a target of racism can have a detrimental influence on multiple aspects of life, such as health (Franklin, 2016), a sense of belonging (Lewis et al., 2021), and career progression (Bhopal, 2020). It is important to investigate intervention strategies for racially motivated bullying in higher education settings to increase staff and student well-being and inclusivity in the campus environment.

Bystanders, individuals who are present in (or knowledgeable of) situations are potentially a powerful source of help in tackling race/ethnicity-based racism in higher education. Bystander behavior has been defined as “Action taken by a person or persons (not directly involved as a target or perpetrator) to speak out about or to seek to engage others in responding (either directly or indirectly, immediately or at a later time) against interpersonal or systemic racism” (Nelson et al., 2011, p. 265). Bystanders could help by directly addressing the situation, delegating help to others, delaying help, covertly distracting the situation, or displaying discomfort and disapproval (e.g., York et al., 2021). Bystander behavior might not only stop the racist action but also function as a tool for changing social norms around what is acceptable (Nelson et al., 2011). However, bystanders often have barriers that may prevent them from helping the targets of racism.

### Facilitators and Barriers to Bystander Intervention

Bystander facilitators and barriers have been investigated widely especially in sexual violence (e.g., Mainwaring et al., 2023), with much less empirical literature on racism (Jenkins et al., 2024). Pioneers in this field, such as Derald Wing Sue (e.g., Sue et al., 2020), have suggested several barriers to anti-racist bystander action. Some of these include ambiguity of situations; lack of understanding of what racism is; diffusion of responsibility; fear of repercussions; conflict avoidance; social norms; interpersonal relationships; group membership; or an inability to act because the person feels paralyzed or does not know what to do (Murrell, 2020; Nelson et al., 2011; Sue et al., 2019, 2020). However, many of these factors have been synthesized from theories or other existing knowledge rather than from empirical literature. Data-driven explorations of facilitators and barriers are thus needed to gain a better understanding of bystanders’ behavior when witnessing racism.

Additionally, it has been suggested that the facilitators and barriers to bystander action in racism are highly context-specific (Nelson et al., 2011). However, as in other bystander literature (e.g., sexual violence; Mainwaring et al., 2023), investigations in non–English-speaking countries and diverse settings are rare. In contrast, most research on bystanders in racism has taken place in the United States (Nelson et al., 2011), which has a very specific and peculiar historical, cultural, social, and political context (Zell & Lesick, 2022). To develop effective intervention programs, it is important to understand what the barriers to help are in diverse contexts.

In the present study, we are interested in broadening the horizons by investigating the bystander role in racism in higher education in Finland, a relatively ethnically and culturally homogenous country in Northern Europe. Finland, among other Nordic countries, has an ideology of exceptionalism (i.e., the perception that the country is equalitarian and discrimination does not exist), which can perpetrate racism and make it difficult to recognize (e.g., Clarke & Vertelyté, 2023). Alongside the Europe-wide increase in right-wing populist politics (e.g., Kende & Krekó, 2020), also in Finland, racist attitudes have increased steeply in a relatively short period of time (Penttilä, 2020). Institutional and interpersonal discrimination is evident in schools (Hummelstedt et al., 2021; Zacheus et al., 2019), access to university education (Riitaoja et al., 2022), and “normative whiteness” in higher education experiences of students racialized as non-white (Souto & Lappalainen, 2025). The unique context of racism in Finland could influence several bystander barriers. On the one hand, Finnish exceptionalism could mean that bystanders fail to act because they negate the existence of racism and cannot recognize it. On the other hand, the evident and often open racism could produce the impression that it is a social norm, which could also present a barrier. To develop effective interventions to tackle racism in higher education in Finland, we need to investigate the barriers and facilitators that bystanders may have in this context.

### The Current Study

The aim of this pilot study is to examine students’ perceptions of facilitators and barriers to their intention to intervene in hypothetical situations of racism that take place in higher education in Finland. We acknowledge that the concepts around race, ethnicity, and racism in Finland are highly contested (see, e.g., Souto & Lappalainen, 2025). In this research, we opted to use the concepts of “ethnicity” and “race” in a broad sense when discussing racism (Braveman & Parker Dominguez, 2021). As our study can be considered as a pilot in the context of Finland, we wanted to examine the bystander perceptions of discrimination in a broader sense (i.e., based on their interpretation of the target’s ethnicity/racialized identities).

Because we wish to gain a deeper understanding of bystander behavior in a new context, it is beneficial to utilize an inductive qualitative approach (Labhardt et al., 2024; Robinson et al., 2022). It has been suggested that bystander facilitators and barriers are likely to vary significantly in different social and cultural contexts, warranting an emic, bottom-up approach when initiating investigations in a new country (Lyons et al., 2024a, 2024b). In this pilot study, we will explore bystander responses to vignettes depicting racist incidences in the higher education context in Finland.

## Method

### Participants and Procedure

Participants (*N* = 649; *M*_age_ = 25.70, *SD* = 6.68; 78.6% women, 16.3% men, 3.9% nonbinary, 1.2% did not want to specify their gender) were recruited from the student population of Finnish higher education institutes through mailing lists of student organizations between April and May 2023. Most participants identified with the ethnic/cultural majority group of the country (84.4%). Arts and humanities (24.2%), natural sciences and mathematics (20.2%), and health and welfare (19.0%) were the most represented fields of study.

### Procedure and Materials

The study was administered via the Qualtrics platform in Finnish and English (only 13 responses were in English). We presented the participants with two vignettes depicting a hypothetical situation of racism in the higher education context. The vignettes were created for this study with the help of a small advisory panel recruited through a student organization of students of ethnic minorities. The vignettes were a result of discussions between the research team and the student panel, as well as reading newspaper/magazine articles that interviewed students about their experiences of racism in higher education in Finland. The vignettes depict realistic scenarios of racism on the campus. One of the vignettes is between students, and the other one is racism by a lecturer during a class. Both vignettes include a racist remark and a student from a minority background who is visibly upset or offended.

#### Vignette 1

Imagine you are on a lunch break with other students taking the same course. Student Y, who has an immigrant background, tells how she was in an unpleasant situation earlier that day when a passerby called her names because she wore a head scarf for religious reasons. Student X belittles student Y’s experience and wonders if she even has to wear a scarf because she lives in Finland. You notice that student Y is upset about the comment.

#### Vignette 2

Imagine that you are taking a course where students are asked to do a self-assessment as part of the course evaluation. Teacher X gives instructions on self-evaluation and mentions that Finnish students usually evaluate their performance more truthfully because they are more honest than others. Student Y, who has an immigrant background, is sitting next to you, and you notice that they experience the teacher’s comment being offensive.

After reading each vignette, the participants were asked two open-ended questions: “What factors could **prevent** you from intervening in this situation” (i.e., barriers to intervention) and “What factors could **help** you to intervene in this situation?” (i.e., facilitators to intervention). The collected data consisted of altogether 2,318 responses from 649 participants transposed onto an Excel file where each line represented 1 participant with facilitators and barriers for each vignette placed in separate columns. The study was approved by the University of Helsinki Ethical Review Board in Humanities and Social and Behavioral Sciences [2/2023].

### Data Analysis

We used an inductive thematic analysis (TA), an approach that is well-suited for qualitative online survey data with relatively short responses (see Braun et al., 2021, for a discussion). The four team members consisted of two faculty members (a man and a woman, both racialized as white) and two postgraduate students (two women, one racialized as black and the other racialized as white). Collectively, the team has experience of racism as white bystanders; through family members; or direct lived experience of racism as a black person. We acknowledge that our positionalities and experiences could influence our approach to the analyses. The team discussed and reflected on positionalities, recognizing that the diversity of our experiences was also a strength when discussing the data from multiple perspectives.

Given that the responses were relatively brief, we did the coding at a semantic, rather than at a latent level. We used a “codebook” approach to TA (Braun & Clarke, 2021) which uses a structured coding framework for development and documentation of the analysis. In this approach, consensus between coders (e.g., inter-rater reliability) is not used as an indicator of quality. The findings were a result of team effort in attempts to understand the barriers that bystanders face within the context of the study. The analytical process started with the immersion of the whole team (ML, VM, RA, TZ) in participants’ responses while taking notes on potential codes and themes. VM generated the initial set of data-driven codes on a chunk of data (in Finnish) that included responses from 100 participants, and RA did the same on responses from 100 participants (in English, translated from Finnish by ML who is native in Finnish, and fluent in English). ML, VM, and TZ had a face-to-face, and ML and RA an online meeting to discuss and compare the codes. As a result, some initial codes were merged with others, and additional codes were added or deleted, with ML auditing and supervising the process. We initially worked on separate analyses for the two scenarios, as well as barriers and facilitators in the scenarios. However, it soon became clear that there were significant similarities in the responses to both scenarios. In addition, the facilitators (e.g., having courage) were often similar to the barriers (e.g., being fearful or shy). Thus, we developed a coding and theme development took place across the whole dataset.

Following the creation of the coding frame, it was used in a subsequent round of coding of the whole data set by TZ. The themes and subthemes were reviewed by the team to ensure they accurately represented the data. As a result, some themes were refined and subthemes relocated under new themes. In the last stage, we further defined the characteristics of each theme and decided upon their final names. The first author audited the data. The quotes included in the results were translated from Finnish to English by ML, a fluent speaker of both languages. The quotes in the results will be accompanied by a participant number (e.g., P1; P2) and an indication of whether the response was for Vignette 1 (V1), or Vignette 2 (V2).

## Results

We constructed six themes with several subthemes for both the barriers and facilitators for both vignettes. The themes were the following: (i) Perceived self-efficacy to intervene; (ii) Justification and moral reasoning; (iii) Clarity of the situation; (iv) Responsibility and permission to act; (v) Social relationships, support, and presence of others; and (vi) Consequences and impacts of action.

### Theme 1: Perceived Self-Efficacy to Intervene

In this theme, we outline individual-level factors related to participants’ perceptions of their capability to intervene in the situation or their ability to succeed in their attempt to intervene. We constructed three subthemes around these factors: (i) Emotions evoked by the situation; (ii) Personal characteristics/life challenges; and (iii) Knowledge and skills.

#### Subtheme 1: Emotions Evoked by the Situation

A theme where participants wrote about strong feelings and emotions that could potentially rise in the situation. For instance, the presence of shame, fear, and lack of courage were barriers, whereas the absence of shame and fear, as well as courage, annoyance, and anger, were facilitators. According to one participant, they would intervene if they felt “Anger that makes you forget that everyone is staring” (P369V1). Subtheme 1 also included feelings of surprise and confusion, which could result in a “freezing,” for example, “It is difficult to act when feeling confused” (P227V1); “If I was too upset or confused about how person X reacted in the situation I would not know how to intervene at that exact moment,” (P357V1); or “If I was too shocked to get a word out of my mouth” (P2V2). Students felt that they would be less inclined to act if the situations took them by surprise, leading to paralysis.

#### Subtheme 2: Personal Characteristics/Life Challenges

This subtheme encompasses more stable factors, such as personal traits (e.g., shyness), or struggles with mental health or other demanding life situations. Many wrote about how they felt depressed or anxious in their daily lives and did not have the mental energy to help others. For example, one participant disclosed how they have been “. . .diagnosed with a moderate fear of social situations, especially with unfamiliar people. Even if I felt ethical and moral pain from not intervening in the situation, I might just be struggling with my own problems so much that I wouldn’t be able to help” (P390V1). This juxtaposition of social obligation to act and the limits of one’s coping was visible also in the response of one participant who branded the struggles in one’s personal life as “selfish reasons,” writing that “in general, the lack of one’s own wellbeing makes it difficult to intervene in such situations. One may already have enough problems” (P379V2).

#### Subtheme 3: Knowledge and Skills

Participants’ perception of their (lack of) knowledge and skills was mentioned as a barrier and a facilitator. If people thought that they didn’t know enough about the debates around racism, they were more reluctant to help. Also, the perceived inability to come up with reasoned arguments on the spot, could lead to an embarrassing situation for the bystander. One individual wrote how “I’m not good at coming up with good reasoning and reasonable comments out of the blue, which would probably raise the threshold to open my mouth in the situation” (P473V1). Often, participants mentioned how they lacked a toolkit for action. Being prepared in advance could be something that facilitates both knowledge, as well as readiness to act. This could take the form of “Knowing good and easy intervention phrases, so one would not ‘freeze’” (P304V1), or “Experience of how to act in a situation like this, and clear advice from university on what to do” (P493V2). Participants sometimes feared that their help would make the situation worse, for example, “Uncertainty about how to react so as not to be offensive or escalate the situation even further” (P516V1). In turn, increased knowledge and education about racism and diversity were seen as facilitators of bystander action (e.g., “additional training for students on the diversity of people and cultures, and same for teachers”; P520V2). This preparation could take the form of discussions among friends, and also education and campaigns in the university.

### Theme 2: Justification and Moral Reasoning

Under this theme, we constructed three subthemes that were named (i) Justification and arguments; (ii) Morals and values; and (iii) Attributional reasoning. The key component here was how participants justified their behavior using personal values and ideologies. Some fostered racist views, where blaming the target was one of the barriers to help. Others saw intervention as their moral and ethical duty, discussing how nothing could prevent them from providing help.

#### Subtheme 1: Justification and Arguments

This subtheme included justifications for inaction when participants thought that the perpetrator made an argument that had reasonable evidence behind it, for example, “Thoughts on whether the teacher could be right and if they have evidence for the argument” (P106V2). The justifications for helping/not helping were often based on personal opinions of the bystander. For example, “If I were to disagree with the teacher quite a bit. . .not all Finns are honest either, so I would point that out” was mentioned as a facilitator justifying help (P249V2). Justifications that were mentioned as a barrier were blaming the targets of racism, for example, “If Y herself is arrogant and refuses to understand Finnish culture or adapt to it” (P295V1). There were other comments about how the target’s reaction (i.e., anger), or their religious conviction was a factor in bystander action. Responses such as “people should be able to put up with these comments. . .you really don’t have to wear terrorist scarves in Finland” (P530V1) highlighted how the participants’ xenophobic views stopped them from providing help. This subtheme demonstrated that bystanders justify their behavior depending on the reasonableness of the perpetrators’ arguments, and how much they agree with them.

#### Subtheme 2: Morals and Values

This subtheme highlighted how personal values can facilitate bystander behavior. For many participants, intervention related to a core moral value, where a sense of justice dictated the necessity to help (e.g., “Standing behind my anti-racist ideas without shame or fear of others’ reactions,” P640V1; or “Injustice would feel really significant and clear in this situation,” P77V2). Many participants thought that intervening would be self-evident, such as “I know for sure that I would intervene if this happened to me, whether I knew the people or not. I feel that intervening is a matter of ethical responsibility” (P449V1). Bystanders were guided by a desire to “do the right thing,” and helping was seen as obvious for these individuals.

#### Subtheme 3: Attributional Reasoning

This subtheme underscores the importance of participants’ perceptions of whether the perpetrator really meant what they were saying. For some participants, lack of deliberate racism was a barrier to intervention, whereas to others, it was a facilitator. In addition, perceiving the situation as an example of blatant racism was both a facilitator and a barrier, depending on the individual. Unintentional racism was mentioned as a barrier by several participants in statements such as “I assume that student X does not understand that she has hurt another’s feelings” (P313V1) or “If person X formulates their question politely in a way that shows genuine interest and curiosity, and if person Y answers the question briefly and lightly and the conversation moves forward naturally” (P371V1). On the contrary, some participants were *more* likely to intervene if they thought that racism was unintentional, demonstrating how a lack of bad intentions could also function as a facilitator. Help was also facilitated if the participant perceived the racism as clear and obvious, for example, “The more blatant the act, the easier it would probably be to intervene in the situation (lower risk of misinterpretation)” (P642V2). These responses show how complex bystander behavior is, where the actions are likely to be guided by multiple factors that could affect how individuals perceive the situations.

If the racist remarks were considered humorous rather than malicious, the threshold to provide help was higher. For instance, if the perpetrator was “. . .using humour in the teaching situation, the meaning of their comment would not be so strong” (P397V2). The perpetrators’ (old) age was also used as an excuse for failing to help (e.g., “If the teacher is really old and I would think that they will die soon,” P132V2). There were several mitigating factors that participants used to justify their bystander decisions, varying from racist ideologies and morality to blaming the victim and excusing the perpetrator.

### Theme 3: Clarity and Constraints of the Situation

Theme 3 presents barriers and facilitators that relate to situational factors that might influence how clearly participants see, hear, and understand the situation. This theme takes into consideration factors that influence the possibility of being heard; the possibility that the bystander does not understand the situation because of situational factors (e.g., chaos, noise); inattention of the bystander; lack of clear nonverbal cues that indicate racism; or not seeing the situation from the start. We divided this theme into two subthemes: (i) Situational constraints and (ii) Factors that relate to understanding of the situation.

#### Subtheme 1: Situational Constraints

In this subtheme, participants speculated how the place of the incidence (including noise/chaos) could impede understanding of the situation. Participants wrote how they may not get an opportunity to speak if there are many people in the situation, or how their voices may not be heard if they tried to speak (e.g., “Presence of a lot of people, and not getting an opportunity to speak,” P505V1, and “If I would not get a turn, because everybody were talking so much that I wouldn’t be able to get my voice heard. I can’t think of another obstacle,” P364V2). The lecture theater itself could be a situational constraint, for example, “If it was a really big lecture hall where I would almost have to shout to get my point heard or if I wasn’t sure what happened” (P304V2). Sitting in close vicinity (i.e., at the same table or near the target of racism) was also mentioned as a facilitator, “If I was sitting close to both Y and X, it would be easier to intervene in the situation” (P381V1), and sitting far away was a barrier.

#### Subtheme 2: Factors That Relate to the Understanding of the Situation

In this subtheme, we included responses where participants wrote about certainty of racism if they did know the full situation. Participants wrote about not having a full picture due to not paying enough attention, for instance, “If I wasn’t awake in the conversation, but I was thinking about my own stuff. It could register after the situation is over, and I might not be sure if I understood the situation correctly” (P640V1). Not hearing the whole conversation was a barrier, but even then, understanding the nonverbal cues could overcome the barrier, “If I hadn’t heard the whole conversation between X and Y, I might hesitate to intervene. But even then, I would ask what’s happening, if I would definitely see that Y was upset about something” (P643V1). In a similar way, participants talked about hearing/seeing the whole situation, and understanding what is going on as a facilitator to help.

### Theme 4: Responsibility and Permission to Act

We constructed theme 4 around participants’ feelings of personal responsibility, divided into two subthemes: (i) Permission to help/need to act, and (ii) Responsibility to act.

#### Subtheme 1: Permission to Help/Need to Act

Responses in this subtheme included the target’s direct (i.e., verbal) or indirect (i.e., nonverbal) signals. These were mentioned both as barriers (i.e., when the target does not signal in any way that they would like to be supported) and facilitators (i.e., when the target sends a clear signal that they would appreciate help). Participant wrote how “If person Y seems like she doesn’t want to make a big number at the time of the incident, I wouldn’t take the conversation further” (P134V1), and “I wouldn’t be sure what the offended person would think if I intervened while the whole class is listening” (P612V2). Often, the participants mentioned clear verbal instructions from the target as barriers (e.g., “Person Y asks me not to interfere with whatever person X says and does,” P12V1) or facilitators (e.g., “If Y themselves express that they want help,” P502V1).

If participants had the impression that the target could defend themselves, they thought that intervention was not necessary. For example, participants wrote how they would not interfere if there was “. . .a wish of the target that I do not interfere. For example, they may want to justify themselves and guide the conversation. Then I could be there for them in other ways” (P70V1), or “I wouldn’t be sure what the offended person would think if I intervened when the whole class is listening” (P622V2). Indeed, some discussed how they did not want to offend the target by escalating the situation, “If I knew that person Y doesn’t want me or others to intervene, for example, because of the fear of the situation escalating, or because they don’t need a ‘white saviour’” (P460V1). Having a “permission” of the target was perceived as a facilitator to bystander help. This permit could come from verbal or nonverbal signals, or knowing the target from previous interactions. Participants wrote how they would intervene if “. . .I knew person Y well and could be sure that they would like my help, and would not feel that I am trying to signal virtue to others at their expense. . .” (P477V2), or “If person Y clearly shows her feelings and I am ‘sure’ that they would like someone to intervene, regardless of what person X says and does” (P416V1). These responses demonstrate how bystanders are more likely to intervene if they have clear indications that the target of abuse gives their consent to the help.

#### Subtheme 2: Responsibility to Act

This subtheme was about feeling responsible to end the situation (e.g., other people did not act). In a similar way, if other people had already intervened, or if the situation ended quickly, participants thought that there was no need for their help. For instance, one participant wrote that a barrier is if “Someone else intervenes faster and louder before me” (P441V1), and another mentioned how “if there are already many people in the situation who are having a constructive discussion on the topic and I would feel that my ‘contribution’ is not needed” (P452V1). Participants indicated how they wanted the abuse to stop, but felt no need to contribute to help if others would take action.

### Theme 5: Social Relationships, Support, and Presence of Others

This theme is an overarching theme for how individuals, their relationships, and support mechanisms aid or prevent bystander behavior. We constructed four subthemes within this broad theme, which we named (i) Presence of others; (ii) Support mechanisms; (iii) Conformity and social norms; and (iv) Personal relationships and hierarchical position.

#### Subtheme 1: Presence of Others

This subtheme was often mentioned in one word or in a short sentence, and it was not possible to draw deeper conclusions for why it was the case. The same person could mention both the presence and absence of others as a barrier (e.g., “If there were only me, X and Y in the situation. Or if there were a LOT of other people in the situation,” (P31V1); or “If there is no one else in the situation but me, Y and X, I’m sure I would intervene in X’s behavior. Or if there are others in the situation who express their support for Y first, it is more likely that my own views will also be expressed at a lower threshold,” (P95V1). Sometimes, the participants explained a little more, linking the presence of others to uncertainty of outcomes, for example, “If I felt that the situation might escalate and there were many students whom I do not know well/whose reactions are unclear” (P325V1). The presence of others was also discussed in terms of causing an unpleasant “scene.” The presence or absence of others could be linked to several other factors that depend on the individual and the unique circumstances.

#### Subtheme 2: Support Mechanisms

Here, we included comments about whether other people did or did not join in with the bystander to help, and whether the university has mechanisms for reporting racism. This was both a barrier (i.e., “If the university didn’t have a channel to report harassment or discrimination,” P597V1) and a facilitator (i.e., “University’s clear instructions on how to act in such situations and who to tell about it,” P402V2). Participants also wrote about their hesitance to help if they had not been supported in the past when trying to report racism. Having clear rules around anti-racism; intervention workshops and campaigns; and anti-racist curriculum were all mentioned as facilitators.

#### Subtheme 3: Conformity and Social Norms

Consisted of responses that mentioned the general atmosphere around tolerance of racism, and the behavior and reactions of others. For example, if the participants felt like they were the only person taking the side of the target of racism, they would be reluctant to act. Also, another barrier was the impression that everybody else supported the perpetrator of racism, for example, “If most people side with X, or behave belittlingly toward Y.” (P29V1), or “It could be potentially difficult to intervene in the situation if there were several friends/supporters of the perpetrator. It could also be difficult if I was alone, surrounded by people who felt it is pointless to intervene” (P158V1). Participants wrote how they gave up to the group pressure because they were afraid that the group would turn against them.

#### Subtheme 4: Personal Relationships and Hierarchical Positions

This theme was around how interpersonal relationships (whether people in the situation were familiar to each other), as well as power relations, prevented or facilitated intervention. For instance, a barrier was if “The person in question is an authority for both of us, and I have reason to suspect that my intervention would cause resentment to someone else besides myself” (P12V1). In addition, social relationships either hindered or facilitated help. Some of the barriers and facilitators that were mentioned in this context were if the participants knew (e.g., “If I knew teacher X well, it would be easier for me to intervene in the situation. If I didn’t know them well, then clear, easy-to-find and reliable reporting channels would make it easier to intervene. . .,” P630V2), or did not know the perpetrator personally (e.g., “If I did not know the teacher—it would be easier to say no to a stranger,” P31V2). Knowing the target was discussed in terms of a facilitator (e.g., “If I knew the student in question, I would know that she hopes that such situations will be dealt with publicly,” P501V2). Also, a barrier was if the perpetrator and the target seemed familiar with each other. One participant wrote how “If people X and Y are closer to each other than I am to either of them. If I don’t know anyone well” (P591V1) would be a barrier to help.

Likes and dislikes were also relevant. If the participants liked, respected, or admired the perpetrator and disliked the target, intervention was deemed as more difficult. P232V2 wrote how it would be a barrier if “The teacher had been really nice before, or I personally didn’t like student Y. Maybe they ate my lunch and crushed my bike with their car, and so on.” Liking the target, and disliking the perpetrator was a facilitator.

### Theme 6: Consequences and Impacts of Action

In the final theme, we discuss the consequences and impacts of action in terms of (i) Fear of negative consequences and (ii) Possibility for change.

#### Subtheme 1: Fear of Negative Consequences

Participants discussed multiple potentially negative consequences that would stop them from intervening. These consequences were around physical aggression and fear for one’s safety; social consequences (e.g., losing status, friends, gaining a reputation, being a future target); or academic consequences (e.g., grades and future employment). Many times, participants just wrote “fear of negative consequences” without specifying what these might be.

The social consequences were mentioned by one participant who wrote how “. . .social consequences of ‘rocking the boat’ in university circles, and potentially being targeted if the information about the situation spreads far” were intervention barriers (P28V1). Being socially ostracized, bullied, or having a reputation was a concern that was mentioned as a barrier in both vignettes.

The potential for escalation and violence were also negative consequences that were mentioned as barriers, and the lack of these consequences were facilitators. For instance, participants wrote that “Threat of violence on behalf of person X, or other threatening atmosphere” (P303V1) or “. . . if teacher X was prone to aggressive behavior or speech” (P81V2) were barriers. On the other hand, comments such as “. . .if I thought it would be safe to intervene, i.e. if person X wasn’t too aggressive. . .and I would talk to person Y later and support them” (P21V1) demonstrated how feeling safe would facilitate intervention.

The fear of academic and employment consequences was an important barrier. In one post, the intersectional position (i.e., own ethnic minority status) could have influenced both the fear of negative social and academic consequences: “The fact that I would know that the teacher of the course influenced my academic success with their power, as well as the fact that the students around me are all white Finns. It’s tough being the ‘woke-poc’ that others find annoying” (P86V2). Fear of retaliation could also be related to the position of authority, such as “If the teacher in question is in a strong position of authority, I know they will ‘retaliate’ somehow, e.g. by giving a lower grade. If the teacher is scary” (P337V2).

#### Subtheme 2: Possibility for Change

Here, we included comments that highlighted how the perceived impact of the intervention influenced bystander decisions. For example, if participants thought that the perpetrator would not change their opinion, intervention was seen as more difficult, such as “If I knew that the teacher has a difficult character, is always right, and refuses to change their opinion. In this situation I would find it pointless to defend person y because person X is unlikely to change his position” (P303V2), or “If I knew that person X was not going to change her behavior despite the intervention” (P611V1). Help would be easier if the perpetrator “. . . could evaluate their own actions based on the feedback” (P95V2). Possibility of change was also discussed in terms of the university/staff taking complaints seriously and acting on them in a way that results in a change.

## Discussion

This study contributes to the sparse knowledge of bystander behavior in racism in the higher education context in Finland, suggesting multiple interlinking barriers and facilitators. Participants mentioned diverse cognitive, affectual, circumstantial, and structural/hierarchical factors, which were similar to findings from other countries (see Nelson et al., 2011; Sue et al., 2020). Below, we discuss the results with a reference to previous research, and the unique context of Finland.

In theme 1, we identified individual-level factors around perceived self-efficacy (i.e., situational emotions, personal characteristics, life challenges, and perceived skills) that both facilitate and prevent bystanders. As a barrier, self-efficacy was related to shyness, social anxiety (see also Brewer et al., 2024), and “freezing” due to a shock. In addition, self-efficacy was associated with strong emotions, which were both a barrier (e.g., shame and fear) or a facilitator (e.g., anger, empathy; or the lack of shame and fear). These emotions could have links to other factors such as shame in the presence of others (e.g., Yule & Grych, 2020); anger and empathy linking to social justice attitudes (e.g., Selvanathan & Lickel, 2019); or the need to outwardly express one’s anti-racist values through showing anger (Hyers, 2007). Because of the brevity of many of the responses, it was not possible to analyze further exactly why emotions were important.

Self-efficacy was also related to current life challenges. This has been discussed previously in terms of “low emotional reserve” (O’Brien et al., 2023). When emotional reserves are depleted (e.g., bystanders are hungry, stressed, tired, busy, depressed, etc), responding to racism in real time is challenging. Perhaps these barriers could be overcome if individuals perceived bystander behavior as a collective responsibility where all members of the community have the duty to help, irrespective of their momentary emotional reserves (see also Robinson et al., 2022).

As well as affective elements, meta-cognitive perceptions of one’s own knowledge and skills were relevant. Lack of knowledge on how to act (Hoxmeier et al., 2020), as well as limited knowledge of what racism is (Wong et al., 2021) have been identified as bystander barriers in previous research. Increasing awareness and skills could improve bystander willingness to help in the context of racism in Finland too, especially as people generally do not have much knowledge of the topic (Mkwesha & Huber, 2021).

Under theme 2, “Justification and moral reasoning,” participants justified their inaction by blaming the targets, excusing the perpetrators, and referring to freedom of speech. The idea that everyone is entitled to their opinions has been discussed as a barrier in previous publications (e.g., Nelson et al., 2011). However, this has received surprisingly little empirical attention. The freedom of speech argument is used to deny racism in Finland, which could lead to victim blame (Pettersson, 2020). This could make bystanders unsure about whether it is in their place to intervene. Indeed, as well as mentioning freedom of speech, participants wrote about how they would not help if the target reacted strongly or somehow invited the racist abuse to themselves. Blaming the targets of racism for being insensitive is a secondary microaggression that can occur when the targets respond to racism (Johnson et al., 2021). Victim blame has been identified as a prominent bystander barrier in sexual violence in English-speaking countries (Mainwaring et al., 2023). It appears that victim blame also plays a role in Finland, in the context of racism. In terms of educational suggestions, it may be useful to develop bystander interventions that aim to increase empathy, potentially via intergroup contact (Abbott & Cameron, 2014). Increasing empathy could reduce victim blame and justifications for perpetrator’s behavior, leading to higher bystander readiness to intervene (Wachs et al., 2023).

Other justifications excusing the perpetrator were deliberateness of racism. When the perpetrator had mitigating factors (e.g., old age and unintentional racism), intervention was not deemed necessary. The lack of intentional racism as a barrier could be connected to the avoidance of embarrassing situations where the perpetrators are accused of something that they were not consciously aware of. Hinting that a person might behave in a racist manner evokes strong emotions not just in perpetrators, but also in other (white) bystanders (Sue et al., 2020). Erring on the side of caution could be a way of avoiding embarrassing social situations in Finland, where white people are socialized to “racial illiteracy,” the unwillingness to understand issues with race and ethnicity (Mkwesha & Huber, 2021). On the other hand, there were individuals who were always willing to intervene, even when they thought that racism was unintentional. Moral arguments and values were strong justifications for helping. Indeed, many mentioned how intervention was self-evident, more like a moral duty. This indicates that some of the participants identified as allies, people who recognize racism and are willing to act to call it out (Williams & Sharif, 2021).

Under theme 3, we discussed situational constraints and clarity of the circumstances. Participants wrote how they would not act if they were not sure what had happened, which could be because they did not see/hear the event fully for various reasons. Situational ambiguity has been identified as a bystander barrier in other reviews and empirical studies (see Sue et al., 2020), and could link to multiple other factors. For instance, if the cultural norm (i.e., contextual factor) is to “stay quiet,” individuals who have a need for cognitive closure (i.e., individual factor) could avoid intervening because they are uncertain about what is happening (Gelfand & Jackson, 2016). Indeed, previous bystander studies have failed to link the situations with contextual (e.g., social norms) and individual-level factors (see Mainwaring et al., 2023). To develop a well-rounded picture of bystander barriers in racism in Finland, future studies could try to entangle the relationships between situations, contexts, structures, and individual differences.

We created theme 4 around perceived responsibility and permission (from the target) to act. Participants wrote how they would intervene only if the target gave their permission, either directly, or via indirect nonverbal communication. If participants considered that the target could handle the situation without help, they were hesitant to help as bystanders. Some wrote how an unwanted intervention could be interpreted as them wanting to be a “white saviour,” adding to racism rather than working against it. Racialized minorities’ input in intervention development is crucial to develop skills in assessing when intervention is welcomed (Selvanathan et al., 2023).

In theme 5, the complexity of the social relationships between the bystander, perpetrator, target, and other bystanders highlighted how diverse the responses depend on the individual and their circumstances. For instance, presence and absence of other people could both facilitate and impede help (see Mainwaring et al., 2023, for similar quantitative findings in sexual violence context). The same was true for interpersonal relationships—for some participants, knowing the perpetrator personally was a facilitator, whereas for others, it was a barrier. The bystander-perpetrator relationship has been investigated within the “social control” framework (e.g., Moisuc & Brauer, 2019) which proposes that bystanders can reinforce social norms by intervening in norm violations. Depending on the context, bystanders may be more likely to exert control (i.e., intervene) when the perpetrator is a friend (Moisuc & Brauer, 2019), or similarly, when the perpetrator is a stranger. The diverse responses in our findings could connect to how individuals perceive social norms in a country with highly polarized views on issues such as immigration (Rovamo et al., 2023). The potential associations between interpersonal relationships and social norms should be investigated further in future studies.

In theme 6, “Consequences and impacts of action,” participants wrote about multiple negative (e.g., social, physical, and academic) consequences because of intervention (e.g., gaining a reputation for being a difficult person; see also Xie & Galliher, 2023). In addition, intervention was facilitated by the perception that it results in a positive change and prevented by the perception that it would not have any impacts (e.g., in the behavior or attitudes of the perpetrator). In this theme, hierarchical and power imbalances were mentioned as barriers especially in the classroom vignette scenario. Racism from the part of teachers is often unchallenged due to fear of retaliation (e.g., giving lower marks), which could lead to adverse academic consequences. Indeed, one of the barriers under theme 5 was the availability of institutional support to the bystanders, who were less likely to intervene if the university lacked structures to facilitate anonymous reporting. Thus, for bystanders to act in racism from the teacher, anonymity is a key facilitator. The higher education institutions in should work toward ensuring that concerns about racism are addressed effectively, and preferably via anonymous routes for the bystanders. More work is needed in Finland to challenge the existing power structures, and aid students to be active anti-racist bystanders (see also Alemanji & Mafi, 2018).

Our study is not without limitations. One of the largest constraints of the results was that the responses were sometimes quite short and would have benefited from more elaboration. As an example, when participants wrote about “presence of others” as a barrier, it was not clear whether this linked to the idea of going against social norms, or if it could be interpreted as pluralistic ignorance, or something else. In interview settings, answers like these could be elaborated further with additional questions. Qualitative online surveys have been critiqued for rigidity and lacking richness of data (Braun et al., 2021). However, this approach has also been praised because it can reach diverse populations, as well as guarantee full anonymity which could remove socially desirable responses (see Braun et al., 2021 for a discussion). Thus, we think that the online survey method could also be one of the strengths of the study.

In conclusion, our preliminary, descriptive results suggest that in Finland, bystanders face multiple contextual, situational, individual, and structural/hierarchical factors that interact with each other in a complex manner in preventing or facilitating action. These findings could be useful when devising interventions that are effective in higher education in Finland. Generally, bystander behavior in racism would benefit from qualitative investigations, leading to interventions that are directed toward removing barriers unique to each context.
